# PARAFAC Decomposition for Ultrasonic Wave Sensing of Fiber Bragg Grating Sensors: Procedure and Evaluation

**DOI:** 10.3390/s150716388

**Published:** 2015-07-07

**Authors:** Rencheng Zheng, Kimihiko Nakano, Rui Ohashi, Yoji Okabe, Mamoru Shimazaki, Hiroki Nakamura, Qi Wu

**Affiliations:** 1 Institute of Industrial Science, The University of Tokyo, Tokyo 153-8505, Japan; E-Mails: ohashi@iis.u-tokyo.ac.jp (R.O.); okabey@iis.u-tokyo.ac.jp (Y.O.); wuqi@iis.u-tokyo.ac.jp (Q.W.); 2 Interfaculty Initiative in Information Studies, The University of Tokyo, Tokyo 153-8505, Japan; E-Mail: knakano@iis.u-tokyo.ac.jp; 3 Tokyo Metropolitan College of Industrial Technology, Shinagawa 140-0011, Japan; E-Mail: shimazaki@s.metro-cit.ac.jp; 4 Graduate school of Engineering, Kanagawa University, Yokohama 221-8686, Japan; E-Mail: hiroki-nak@kanagawa-u.ac.jp

**Keywords:** fiber Bragg grating, parallel factor analysis, signal-to-noise ratio, ultrasonic wave

## Abstract

Ultrasonic wave-sensing technology has been applied for the health monitoring of composite structures, using normal fiber Bragg grating (FBG) sensors with a high-speed wavelength interrogation system of arrayed waveguide grating (AWG) filters; however, researchers are required to average thousands of repeated measurements to distinguish significant signals. To resolve this bottleneck problem, this study established a signal-processing strategy that improves the signal-to-noise ratio for the one-time measured signal of ultrasonic waves, by application of parallel factor analysis (PARAFAC) technology that produces unique multiway decomposition without additional orthogonal or independent constraints. Through bandpass processing of the AWG filter and complex wavelet transforms, ultrasonic wave signals are preprocessed as time, phase, and frequency profiles, and then decomposed into a series of conceptual three-way atoms by PARAFAC. While an ultrasonic wave results in a Bragg wavelength shift, antiphase fluctuations can be observed at two adjacent AWG ports. Thereby, concentrating on antiphase features among the three-way atoms, a fitting atom can be chosen and then restored to three-way profiles as a final result. An experimental study has revealed that the final result is consistent with the conventional 1024-data averaging signal, and relative error evaluation has indicated that the signal-to-noise ratio of ultrasonic waves can be significantly improved.

## 1. Introduction

Fiber Bragg grating (FBG) sensors used in structural health monitoring have been studied extensively in terms of their long-term structural operation and safety [1,2]. An FBG is a kind of distributed Bragg reflector that reflects light of a particular wavelength. Because of their small size, passive nature, immunity to electromagnetic interference, and ability to directly measure physical parameters such as temperature and strain, FBG sensors have become an important sensing technology even in harsh environments [3]. Meanwhile, an arrayed waveguide grating (AWG) filter has been applied in a high-speed optical-wavelength interrogation system to speedily reflect wavelength shifts of the FBG and Lame waves detected employing this system [4]. The solid state and low energy of the AWG means that the system can be compact and cost effective. Additionally, the AWG filter has been applied as a demultiplexer to precisely interrogate wavelength shifts of multiple FBG sensors [5].

As a monitoring system, the normal FBG sensor with AWG filter has been used to inspect ultrasonic wave signals in the damage detection of composite materials [6]. However, the received waveform is always affected by noise from light sources. In the conventional method, thousands of measurements have to be made under the same condition, and the data averaged to remove noise [7,8]. Therefore, the system can only be applied in limited situations and is difficult to apply to passive acoustic emission investigations. Recently, a phase-shifted FBG, connected with a tunable laser source, was developed to sensitively detect an ultrasonic signal [9,10]. However, it remains necessary to develop effective measures of resolving the described bottleneck problem of multiple measurements for the normal FBG sensor system.

Related to the application of a signal processing method to improve measuring accuracy of FBG sensors, a correlation signal processing was applied for multimode FBG sensors to improve measuring accuracy for strain and temperature applications [11]. A fairly linear characteristic had been obtained for tensile strain, but the temperature characteristic was somewhat nonlinear. In a parallel demodulation of extrinsic Fabry–Perot interferometer and FBG systems, a signal processing algorithm was proposed by the combination of a Fourier transform spectrum and low-coherence interference to reduce the measurement errors [12]. Additionally, as a digital filter, finite and infinite impulse response algorithms were used to improve the wavelength detection accuracy introduced by the applied strain on the FBG sensors [13]. However, the above three references did not discuss the signal-to-noise ratio in detail, even though the signal-to-noise ratio is one of the most important indexes for a signal processing method. With thorough research, up to now, we could not find any published literature about a signal processing method for ultrasonic wave sensing by application of the FBG system, other than the 1024-data averaging method.

As a principal component analysis in psychometrics, parallel factor analysis (PARAFAC) was developed for unique multiway decomposition without additional orthogonality or independence constraints [14]. In chemometrics, PARAFAC has been extensively studied for the analysis of variance, the unique decomposition of sparse fluorescence, and the prediction of amino-N in sugar samples from fluorescence [15]. The PARAFAC model, known as one of the most adequate, robust, and interpretable multiway methods, is suitable for the batch processing of multi-channel signals and multi-dimensional analyses. The model is receiving interest in the scientific and engineering fields for the analysis of large-scale, arrayed, mixed, or partially missing data requiring increasing computational complexity.

In electrophysiology, PARAFAC has been used to decompose the time-variant spectrum of multichannel electroencephalographic recordings into a series of space–time–frequency components, and thus to search for alpha, beta, and theta activities in a given spectral profile [16,17]. The same method has been applied to reveal significant correlations in electroencephalography and functional magnetic resonance imaging results [18]. As a timely application in engineering, PARAFAC decomposition was adopted for vibration modal analysis of a supported beam in acquiring the natural frequency and modal shape through numerical simulation [19]. For blind speech separation, a PARAFAC-based frequency-domain technique was developed to improve the signal-to-interference ratio, and a low-complexity adaptive algorithm is suited to the under-determined environment in which there are more speakers than microphones [20]. Moreover, recent studies in civil engineering revealed that PARAFAC analysis can be applied to the three-way analysis of structural health monitoring data, blind modal identification of damped systems, and ambient modal identification of a stress-ribbon bridge [21,22,23]. The experimental results also indicated that the PARAFAC model is a preferable denosing method to analyze a hyperspectral image, and a jointly filtering wavelet component tensor was further developed [24,25].

There have, however, been few studies on the application of the PARAFAC model to the signals of optical fiber sensors. A preliminary work was presented in [26], and the present study furthers the work by evaluation of the feasibility of PARAFAC decomposition for the detection of ultrasonic wave signals using optical fiber sensors. In particular, this study continues the development of an integrated signal-processing strategy to improve the signal-to-noise ratio of ultrasonic wave signals, and a comprehensive discussion of the performance of the PARAFAC method is presented for different application conditions.

## 2. Materials and Methods

### 2.1. Ultrasonic Wave-Sensing System

**Figure 1:**
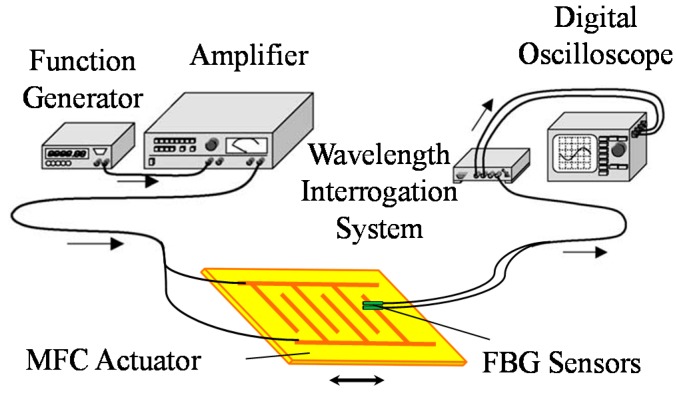
Schematic diagram of the ultrasonic wave sensing system.

The experimental system was an ultrasonic wave-sensing system mainly comprising a function generator, an amplifier, a macro fiber composite (MFC) actuator, two FBG sensors, and a high-speed optical wavelength interrogation system with an arrayed waveguide grating (AWG) filter, and a digital oscilloscope. A schematic diagram of the ultrasonic wave sensing system is presented in Figure 1. An input wave signal generated by the function generator is amplified and sent to the MFC actuator. The MFC actuator used to excite ultrasonic waves is a flexible actuator consisting of thin rectangular piezoceramic fibers sandwiched between layers of an epoxy adhesive and a polyimide film with an electrode pattern [27]. The MFC actuator is bonded to the carbon-fiber-reinforced plastic laminate with an epoxy adhesive.

The FBG sensors glued on the surface of the laminate receive propagating ultrasonic waves. The FBG sensors are fabricated in an optical fiber that has a refractive index that varies periodically along a certain length of the core. When broadband light is injected into the core, the FBG reflects a narrow spectral component at a Bragg wavelength that is proportional to the strain generated by the propagation of ultrasonic waves.

The AWG filter is a kind of planar lightwave circuit that consists of an array of narrow bandpass filters. The signal at each port of the AWG filter is detected by a photodetector, and each pair of adjacent-port signals is processed as a ratio output. When the light reflected from the FBG enters the arrayed filters, the reflection spectrum passes through two adjacent filters. The optical power is then modulated to the ports of the filters, depending on the central reflection wavelength. Since the optical powers can be directly converted into an electrical signal by photodetectors, the AWG filter can detect the Bragg wavelength shift accompanying the high-speed strain changes.

**Figure 2:**
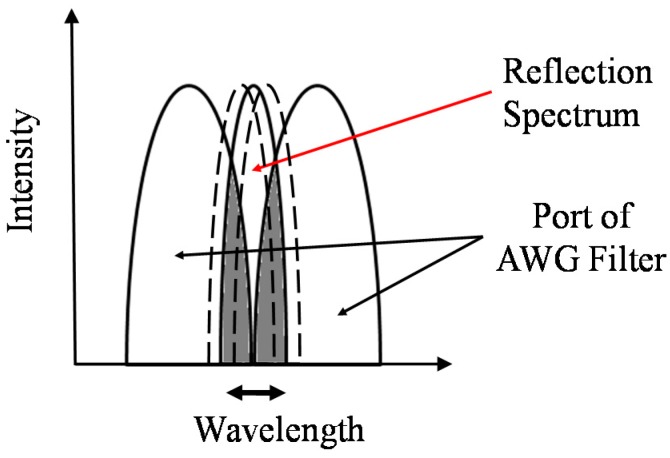
Variation of the reflection spectrum between two ports of the AWG filter.

In this study, two pairs of adjacent ports of the AWG filter are adopted to reflect the wavelength shift from the two FBG sensors. As shown in Figure 2, when the Bragg wavelength of the FBG shifts upon the arrival of an ultrasonic wave, the areas of superposition of the reflection spectrum fluctuate between two adjacent ports of the AWG filter. In this situation, the area of superposition will increase for the port on one side and decrease for the port on the opposite side. Simply put, a significant ultrasonic wave can produce antiphase outputs for two adjacent ports of the AWG filter.

It can be imagined that the measured signal always contains noise from light sources and measuring instruments. On the basis of the hypothesis that noise from light sources interfuses into all ports without a phase difference and that noise from measuring instruments independently interfuses into each port, neither type of noise can result in an antiphase phenomenon. Therefore, a significant signal can be distinguished employing the antiphase feature.

### 2.2. Proposal

**Figure 3:**
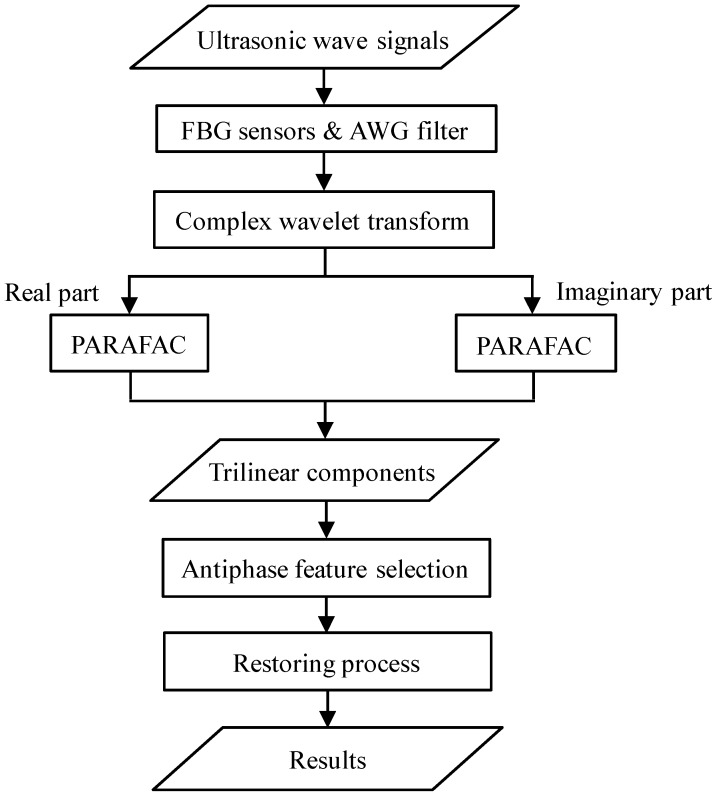
Flowchart of the proposed method.

Figure 3 is a flowchart of the proposed method for the processing of one-time-measured ultrasonic signals. The MFC actuator emits ultrasonic waveforms that are detected by two FBG sensors and transferred to the four ports of the AWG filter. The time history and phase data are then preprocessed by a complex wavelet transform to add frequency data. Afterward, the three-way data are decomposed to multiple trilinear components of temporal, phase, and frequency profiles. Through antiphase feature selection, the target component is restored to the three-way data to give the final result. 

### 2.3. Signal Processing

Wavelet transformations are conducted firstly for temporal data to produce frequency data. Adding relative phase data between adjacent ports of the AWG, temporal data can be converted to a three-way time-varying spectrum array. When *s*(*t*) is considered as the data from each port of the AWG, the wavelet transform is defined as

(1) $$C_{a,b}\text{​ ​}=\int_{-\infty }^{\infty }s\text{(}t\text{) }\frac{1}{\sqrt{a}}\psi \;(\frac{t-b}{a})\;dt$$

where *a*, *b*, and *ψ*(*t*) are the frequency scale, time position, and mother wavelet, respectively [28]. In this report, a complex Morlet mother function consisting of a sinusoid and cosine is used and defined as

(2) $$\psi \text{(}x\text{)}=\frac{\text{1}}{\sqrt{\pi \text{​}f_{b}}}\exp(-t^{2}/f_{b})\exp(\;i\;2\pi \;f_{c}t)$$

where *f_b_* is the bandwidth parameter and *f_c_* is the center frequency. For *f_b_* = 1.5 and *f_c_* =1, when the sample time is *dt*, the relation between the frequency *f* and scale *a* is found to be

(3) $$f=\frac{1}{a\;dt\;}$$

From the transformed data, the three-way data array *S*(*N_f_* × *N_t_* × *N_p_*) can be constructed, where *N_f_*, *N_t_*, and *N_p_* are the numbers of frequency, time, and channel points, respectively. When the wavelet transform of channel *p* at frequency *f* and time *t* is denoted by *C_f,t,p_*, the element of *S*, which is represented by *S_ftp_*, is given by

(4) Sftp=Re[Cf,t,p]

**Figure 4:**
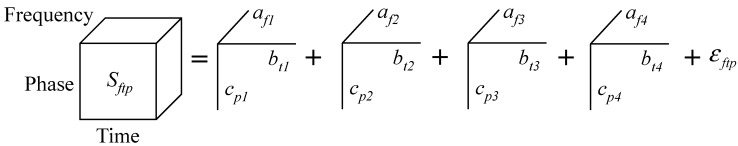
Graphical representation of a four-component PARAFAC model.

PARAFAC decomposition is sequentially conducted for the three-way array *S_ftp_*, which is obtained from the ports of the AWG filter through wavelet transformation, and can be indicated by the temporal, phase, and frequency data array. A four-component PARAFAC model is graphically illustrated in Figure 4.

The three-way PARAFAC decomposition can be characterized by the following generative model

(5) $$S_{ftp}=\sum_{k=1}^{N}a_{fk}b_{tk}c_{pk}+\varepsilon_{ftp}$$

where *a_fk_*, *b_tk_*, and *c_pk_* represent the elements of the vectors of *a_k_*, *b_k_*, and *c_k_*, respectively. Thereby, the three-way data array *S_ftp_* is decomposed into a sum of atoms, where the *k*th atom is the trilinear product of the vectors of *a_k_*, *b_k_*, and *c_k_*, representing frequency, phase, and time signatures, respectively. *ε_ftp_* is the residual that is related to an associated sum-of-squares loss, and the sum-of-squares of the residual array (*i.e.*, ||*ε_ftp_*||^2^) is minimized to find the *N* components (*a_k_*, *b_k_*, and *c_k_*) by using an iterative alternating least squares approach. Within this model, any solution to Equation (5) is a maximum likelihood solution under the assumptions of Gaussian noise [29].

One important problem is to find the appropriate number of atoms *k* when the PARAFAC is applied to data decomposition. A typical approach is to use the core consistency diagnostic, which is known as a simple but powerful tool for finding the appropriate number [30]. The core consistency is defined as

(6) $$Core\text{​​​​ }Consistency=1-\frac{\sum_{f=1}^{N_{f}}\;\sum_{t=1}^{N_{t}}\sum_{p=1}^{N_{p}}{\left(S_{ftp}-T_{ftp}\right)}^{2}}{\sum_{f=1}^{N_{f}}\;\sum_{t=1}^{N_{t}}\sum_{p=1}^{N_{p}}{\left(T_{ftp}\right)}^{2}}\;$$

where *T_ftp_* is the element of the rank-one Tucker model, defined as the equivalent form of a restricted Tucker3 model [31]. When the number of components is appropriate, the core consistency remains around 100%. However, if the core consistency drops noticeably below 100%, it is necessary to reduce the number of atoms in obtaining the appropriate model. Accordingly, the number of components should be chosen according to the core consistency value being close to 100%.

Dependent on the characteristics of frequency, temporal, and phase profiles, the correlation between the input and decomposed atoms can be analyzed to find the target atom with which to deduce the analysis result. Furthermore, the decomposed atom can be restored to three-way data as a PARAFAC-based analysis result. For example, if the *j*th atom is treated as the target atom, its element of the three-way data, *S′_ftp_*, can be expressed by the trilinear product of frequency, temporal, and phase profiles as

(7) $$S\prime_{ftp}=ar_{fj}br_{tj}cr_{pj}+i\cdot ai_{fj}bi_{tj}ci_{pj}$$

where *ar_f_*, *br_t_*, and *cr_p_* are the real parts of the frequency, time, and phase profiles, and *ai_f_*, *bi_t_*, and *ci_p_* the imaginary parts of the frequency, time, and phase profiles, respectively. The real and imaginary parts can be obtained from the wavelet transformation.

### 2.4. Relative Error

As a traditional method, 1024-time measurements made using the FBG are averaged for the noise reduction; the result is referred to as the 1024-time averaged signal. In this study, PARAFAC decomposition is applied to improve the signal-to-noise ratio for the one-time measured signal of the FBG. Therefore, relative errors for one-time measured and restored signals are applied to quantitatively assess the effectiveness of the proposed method. The relative measuring error, *Err*^m^, representing the difference between the results of the one-time measured signal and the 1024-time averaged signal, is defined as

(8) $$Err^{\text{m}}=\frac{\sum_{f=1}^{N_{f}}\sum_{t=1}^{N_{t}}\sum_{p=1}^{N_{p}}\left|S_{ftp}^{o}\text{-}S_{ftp}^{\text{a}}\right|}{\sum_{f=1}^{N_{f}}\sum_{t=1}^{N_{t}}\sum_{p=1}^{N_{p}}\left|S_{ftp}^{\text{a}}\right|}$$

where *S°_ftp_* and *S^a^_ftp_* are the elements of the results of the one-time measured signal and the 1024-time averaged signal, respectively. *N_f_*, *N_t_*, and *N_p_* are the numbers of the frequency, time, and phase points, respectively.

The relative analysis error, *Err*^p^, representing the difference between the results of the one-time restored signal obtained by PARAFAC processing and the 1024-time averaged signal, is defined as

(9) $$Err^{\text{p}}=\frac{\sum_{f=1}^{N_{f}}\sum_{t=1}^{N_{t}}\sum_{p=1}^{N_{p}}\left|S\prime_{ftp}\text{-}S_{ftp}^{\text{a}}\right|}{\sum_{f=1}^{N_{f}}\sum_{t=1}^{N_{t}}\sum_{p=1}^{N_{p}}\left|S_{ftp}^{\text{a}}\right|}$$

where *S′_ftp_* is the element of the results of the one-time restored signal.

## 3. Results and Discussions

### 3.1. Input and Output Signals

**Figure 5:**
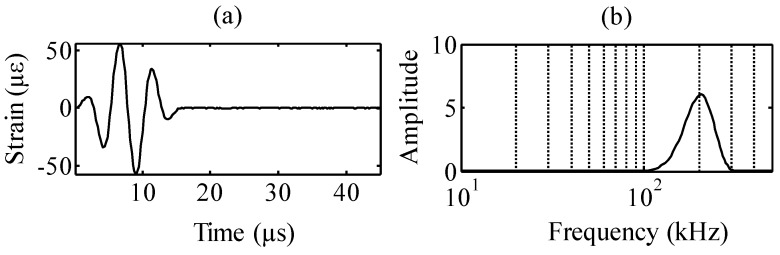
Input wave signal: (**a**) time history and (**b**) power spectral density.

The waveform input to the MFC actuator was a three-cycle sinusoidal wave with a Hamming window. Figure 5 presents the time history and power spectral density of the input signals at a frequency of 200 kHz (Kilohertz, 10^3^ Hz). The sampling frequency is 5 MHz (Megahertz, 10^6^ Hz).

**Figure 6:**
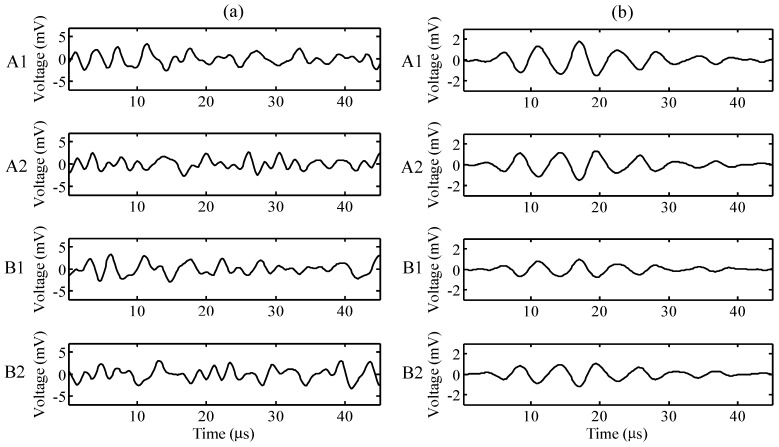
Time histories of ports A1, A2, B1, and B2 for (**a**) the one-time measuring signal and (**b**) the 1024-data averaging signal.

The time histories of the one-time measuring signal are shown in Figure 6a and those of the 1024-data averaging signal are shown in Figure 6b, for the ports A1, A2, B1, and B2. This study applied an arrayed waveguide grating (AWG) to reflect the wavelength shift from the two FBG sensors (see Figure 1). The AWG consists of 40 ports that are arranged in the sequence of the central wavelength, and pairs of adjacent ports from the 40 ports of the AWG can be applied for one FBG sensor. Therefore, for the convenience of distinction in this study, the adjacent ports for the FBG sensor A were defined as the ports of A1 and A2, and B1 and B2 for the other FBG sensor B.

A bandpass filter ranging from 100 to 500 kHz was used for both signals. Under the assumption that the ultrasonic wave signal can be clearly distinguished by employing the 1024-data averaging method, the 1024-data averaging signal was applied as a standard reference in this study. For the one-time measuring signal, the target signals are buried since the amplitude of fluctuation is smaller than the noise. The one-time measuring signal is broadband; therefore, frequency-based filtering is not effective for signal detection.

Because the two FBG sensors were placed at the same position, the four AWG ports should be in anti-phase to each other. Table 1 gives the correlation between signals of ports of the AWG filter, which are processed employing the averaging method and normalization. The correlation coefficient is positive for the signals of ports A1 and B1, but negative for the signals of ports A1 and port A2 or port B2. This means that port A1 is in anti-phase with ports A2 and B2. The same result is confirmed for the signals of the other ports.

**Table 1:** Analysis of correlation between the signals of ports A1, A2, B1, and B2.

Port	A1	A2	B1	B2
A1	1.000	−0.978	0.949	−0.955
A2	−0.978	1.000	−0.950	0.949
B1	0.949	−0.950	1.000	−0.936
B2	−0.955	0.949	−0.936	1.000

### 3.2. PARAFAC Decomposition

**Figure 7:**
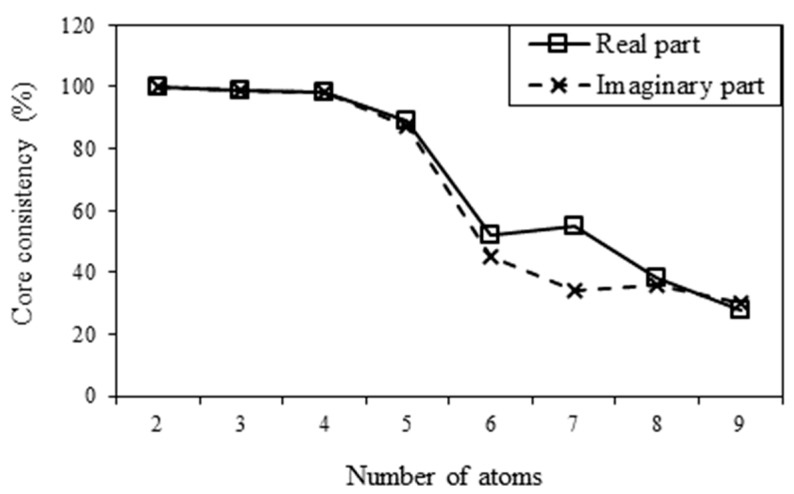
Core consistency for different numbers of atoms.

To decide the appropriate number of components, the core consistency was calculated using Equation (6), after complex wavelet transforms were processed for the one-time measuring signal. For the different numbers of atoms, the results of the core consistency are presented in Figure 7. When there were five atoms, the core consistency values dropped to 89.2% and 87.0% for the real and imaginary parts, respectively. Therefore, there should be four components, for which the core consistency values are closed to 100%.

**Figure 8:**
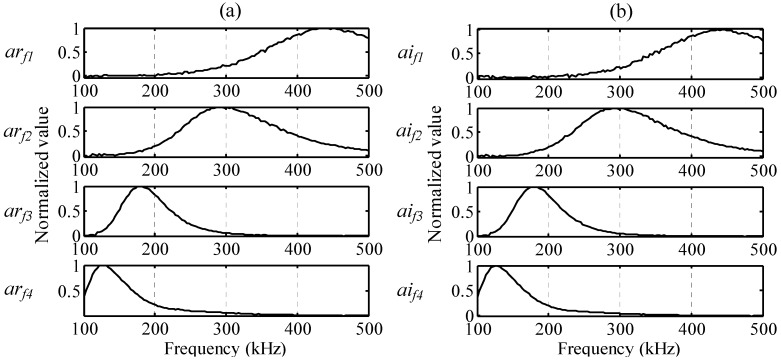
Frequency profiles: (**a**) *ar_f1_*, *ar_f2_*, *ar_f3_*, and *ar_f4_* of the real part and (**b**) *ai_f1_*, *ai_f2_*, *ai_f3_*, and *ai_f4_* of the imaginary part.

**Figure 9:**
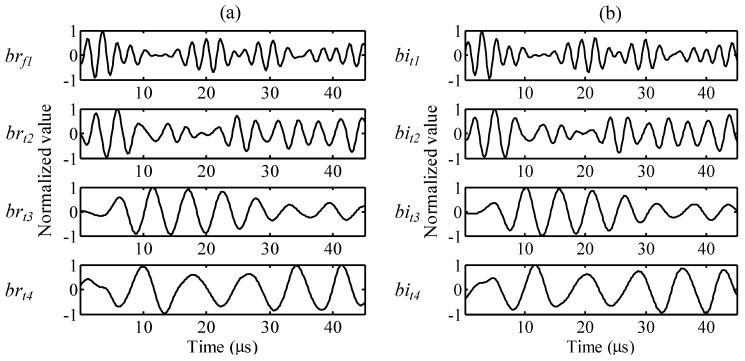
Temporal profiles: (**a**) *br_t1_*, *br_t2_*, *br_t3_*, and *br_t4_* of the real part and (**b**) *bi_t1_*, *bi_t2_*, *bi_t3_*, and *bi_t4_* of the imaginary part.

**Figure 10:**
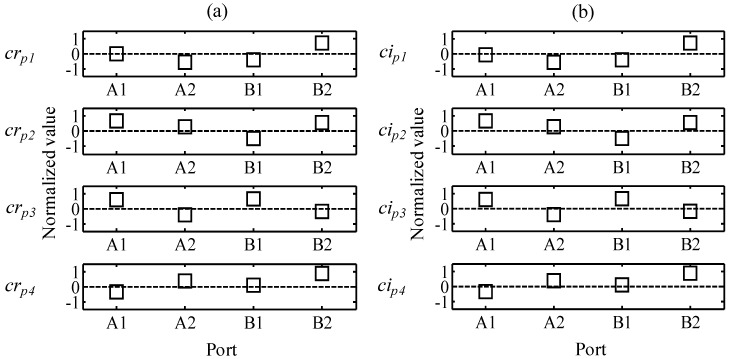
Phase profiles: (**a**) *cr_p1_*, *cr_p2_*, *cr_p3_*, and *cr_p4_* of the real part and (**b**) *ci_p1_*, *ci_p2_*, *ci_p3_*, and *ci_p4_* of the imaginary part.

After complex wavelet transformation, the one-time measured signal is decomposed by PARAFAC. Frequency, temporal, and phase profiles are presented in Figure 8, Figure 9 and Figure 10.

The frequency profiles for the real and imaginary parts are presented in Figure 8a,b. *ar_f_* and *ai_f_* are the frequency distributions of atoms, and the index numbers are the order of the decomposed atoms. The normalized values show the peak frequencies are 449, 289, 179, and 128 kHz for *ar_f1_*, *ar_f2_*, *ar_f3_*, and *ar_f4_* of the real part, respectively, and the peak frequencies are 446, 289, 179, and 125 kHz for *ai_f1_*, *ai_f2_*, *ai_f3_*, and *ai_f4_* of the imaginary part, respectively. The temporal profiles for the real and imaginary parts are presented in Figure 9a,b, respectively. *ar_t_* and *ai_t_* are the time variations of atoms, and the index numbers are the order of the decomposed atoms.

The phase profiles for the real and imaginary parts are presented in Figure 10a,b, respectively. *ar_p_* and *ai_p_* are the relative phases of the AWG ports, and the index numbers are the order of the decomposed atoms. When the reflected wavelength of the FBG fluctuates between the two adjacent AWG ports, the phases of the outputs of the AWG ports are opposite. Thereby, the signs of the relative phase of the AWG ports should be [+ − + −] or [− + − +]. In Figure 10, the elements of *cr_p3_* and *ci_p3_* have the same array of signs as [+ − + −], and thereby, the two elements can be selected as the target atoms with which to deduce significant signals.

PARAFAC can decompose three-way data into the sum of atoms, and each atom can be restored to three-way data separately. Through correlation analysis and feature selection, the target atoms can be restored as significant signals and the other atoms then removed. 

**Figure 11:**
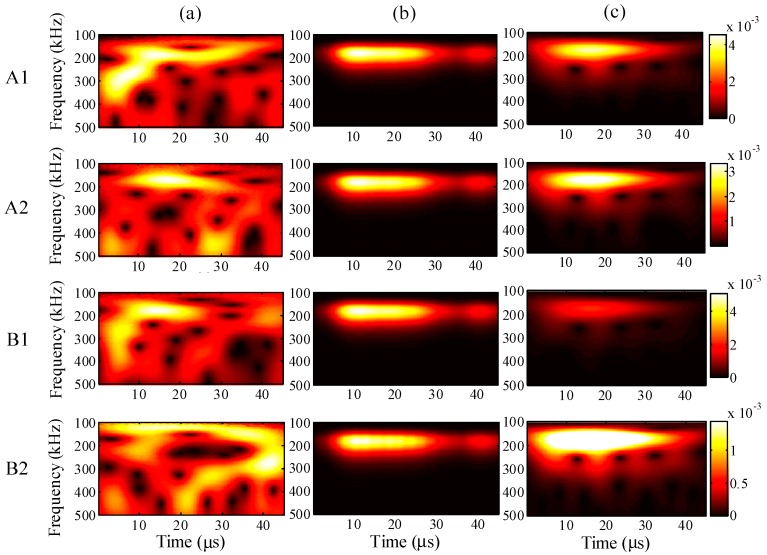
Wavelet transforms at AWG ports A1, A2, B1, and B2: (**a**) one-time measured signal (left); (**b**) one-time restored signal (middle); (**c**) 1024-time averaged signal (right).

When the maximum input amplitude is 58 με, the input period 45 μs, and the input frequency 200 kHz, the wavelet transforms at AWG ports A1, A2, B1, and B2 for the one-time measured signal (left), one-time restored signal (middle), and 1024-time averaged signal (right) are presented in Figure 11, respectively.

For all ports A1, A2, B1, and B2, compared with the 1024-time averaged signal, it is apparent that the one-time measured signals contain much noise. However, the wavelet transforms for the one-time restored signal are clearly around 200 Hz, and the frequency distributions are highly consistent with the results for the 1024-time averaged signal, especially at ports A1 and A2.

### 3.3. Relative Error Evaluation

#### 3.3.1. Input Signal Amplitude

In considering whether the proposed method can notably improve the signal-to-noise ratio of the one-time measured signal, it is necessary to discuss its applicability under different conditions. Therefore, in this study, an experiment was also carried out for different maximum input signal amplitudes, analysis periods, and input signal frequencies. Moreover, relative measurement and analysis errors are assessed according to Equations (8) and (9). The effectiveness of the proposed method is quantitatively evaluated, by comparison of the results of the one-time measured signals, one-time restored signals, and 1024-time averaged signals.

**Figure 12:**
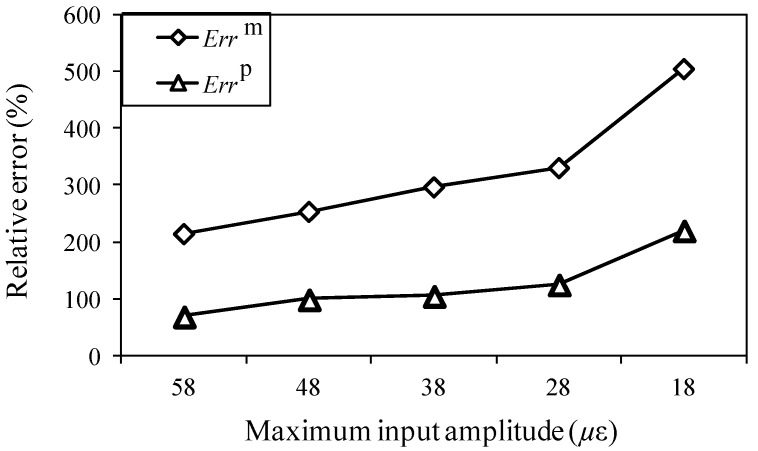
Relative measuring error *Err*^m^ and relative analysis error *Err*^p^ for the different maximum input amplitudes.

For an analysis period of 45 μs and input frequency of 200 kHz, Figure 12 shows the results of the relative measuring error *Err*^m^ and relative analysis error *Err*^p^ at different input amplitudes. The relative analysis errors for the one-time restored signals are clearly lower than the relative measuring errors, which indicates that the signal-to-noise ratio was improved using the proposed method. Additionally, as the maximum input amplitude decreased to 58, 48, 38, 28, and 18 με, both relative errors increased. Additionally, the relative errors sharply increased for the weaker signal of 18 με, and no significant signal was found for the case of 8 με.

**Figure 13:**
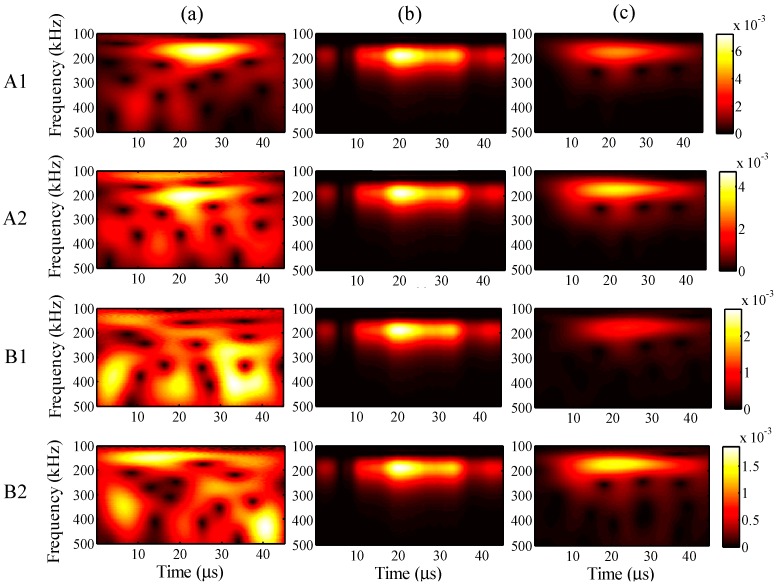
Wavelet transforms for the maximum input amplitude of 48 µε at AWG ports A1, A2, B1, and B2: (**a**) one-time measured signal (left); (**b**) one-time restored signal (middle); (**c**) 1024-time averaged signal (right).

**Figure 14:**
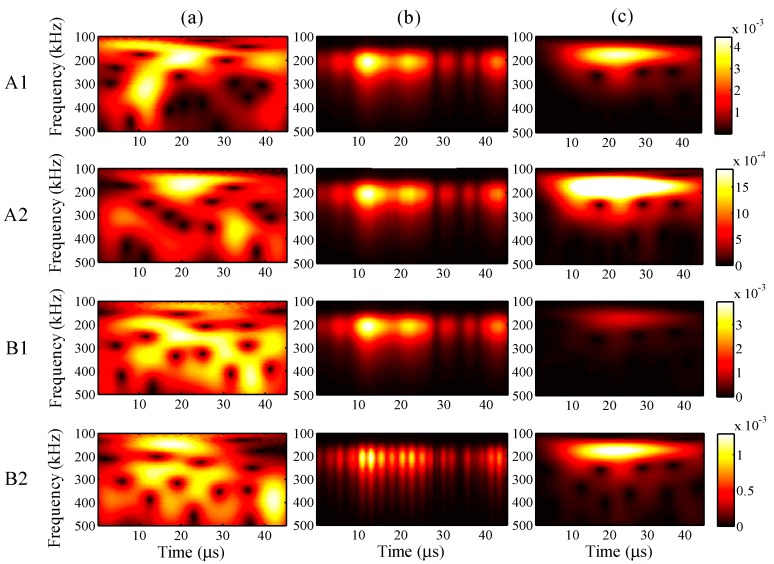
Wavelet transforms for the maximum input amplitude of 38 µε at AWG ports A1, A2, B1, and B2: (**a**) one-time measured signal (left); (**b**) one-time restored signal (middle); (**c**) 1024-time averaged signal (right).

**Figure 15:**
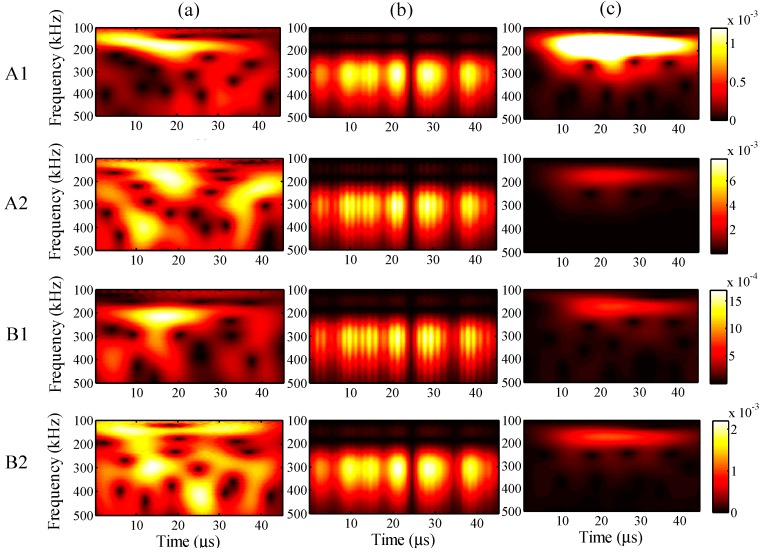
Wavelet transforms for the maximum input amplitude of 28 µε at AWG ports A1, A2, B1, and B2: (**a**) one-time measured signal (left); (**b**) one-time restored signal (middle); (**c**) 1024-time averaged signal (right).

**Figure 16:**
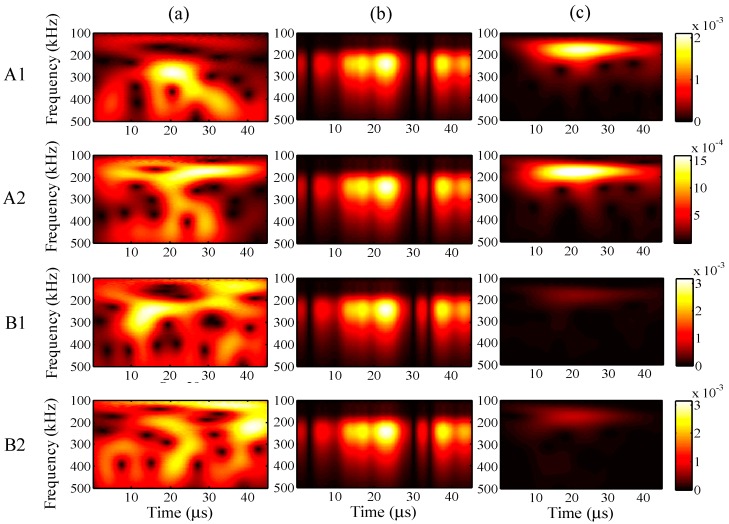
Wavelet transforms for the maximum input amplitude of 18 µε at AWG ports A1, A2, B1, and B2: (**a**) one-time measured signal (left); (**b**) one-time restored signal (middle); (**c**) 1024-time averaged signal (right).

For further understanding of the effectiveness, the frequency distributions of the wavelet transforms were analyzed in all cases. For a maximum input amplitude of 48 µε, the wavelet transforms at AWG ports A1, A2, B1, and B2 of the one-time measured signal (left), one-time restored signal (middle), and 1024-time averaged signal (right) are presented in Figure 13, respectively. Likewise, the results of the wavelet transforms for maximum input amplitudes of 38, 28, and 18 µε are presented in Figure 14, Figure 15 and Figure 16, respectively.

In Figure 13, the results of the one-time restored and 1024-time averaged signals are similar. In Figure 14, however, from the time point of 30 μs, the frequency distributions of the one-time restored signals at ports A1, A2, and B1 begin to disappear in intervals, and similar distributions are observed overall for the one-time restored and 1024-time averaged signals. In Figure 15 and Figure 16, for the one-time restored signals, the frequency distributions are similar at 300 and 250 kHz, which indicates that other signals beyond 200 kHz are mixed in the one-time measured signals and cannot be filtered correctly. For the cases of 28 and 18 με, the relative measuring errors are higher than 300% and it is difficult to obtain a significant signal for the one-time restored signals. In addition, both frequency distributions disappeared in several places.

#### 3.3.2. Analysis Period

**Figure 17:**
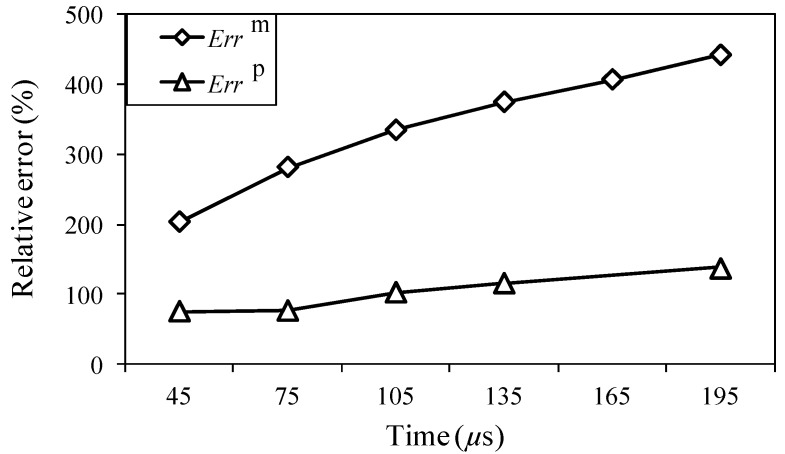
Relative measuring error *Err*^m^ and relative analysis error *Err*^p^ for the different analysis periods.

**Figure 18:**
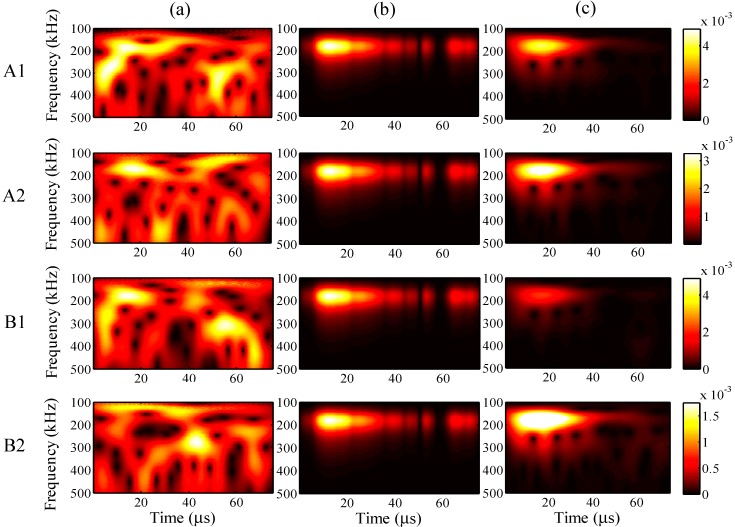
Wavelet transforms for the analysis period of 75 µs at AWG ports A1, A2, B1, and B2: (**a**) one-time measured signal (left); (**b**) one-time restored signal (middle); (**c**) 1024-time averaged signal (right).

**Figure 19:**
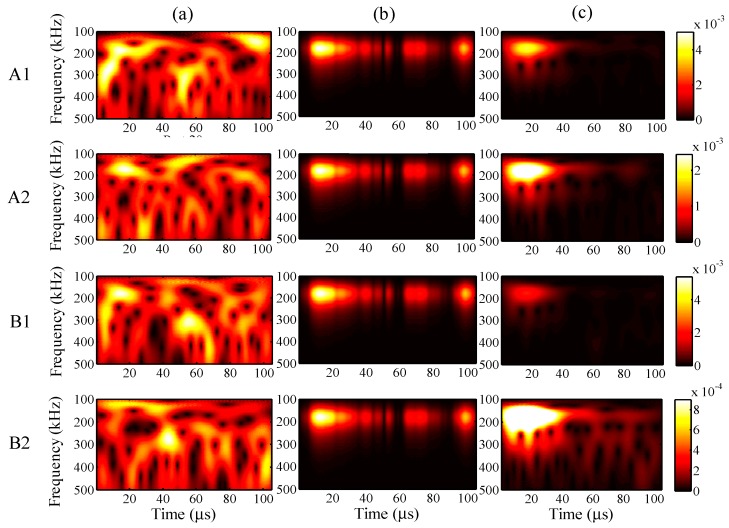
Wavelet transforms for the analysis period of 105 µs at AWG ports A1, A2, B1, and B2: (**a**) one-time measured signal (left); (**b**) one-time restored signal (middle); (**c**) 1024-time averaged signal (right).

**Figure 20:**
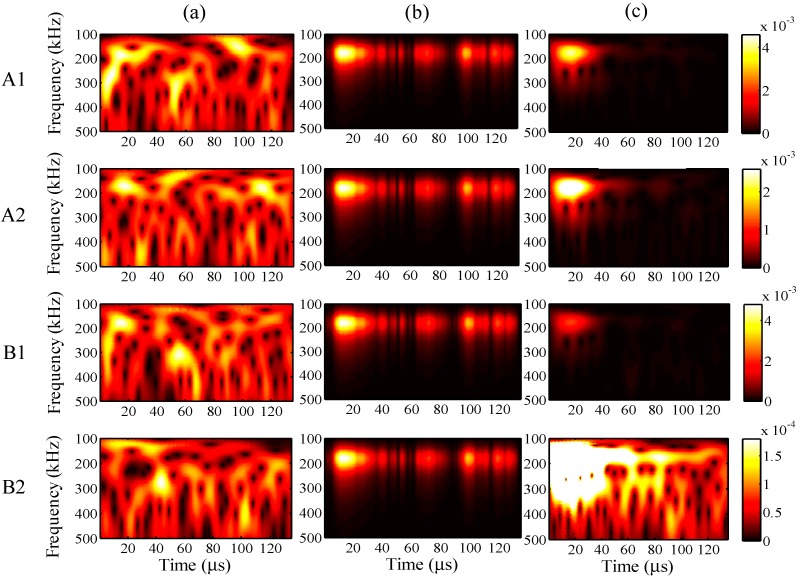
Wavelet transforms for the analysis period of 135 µs at AWG ports A1, A2, B1, and B2: (**a**) one-time measured signal (left); (**b**) one-time restored signal (middle); (**c**) 1024-time averaged signal (right).

**Figure 21:**
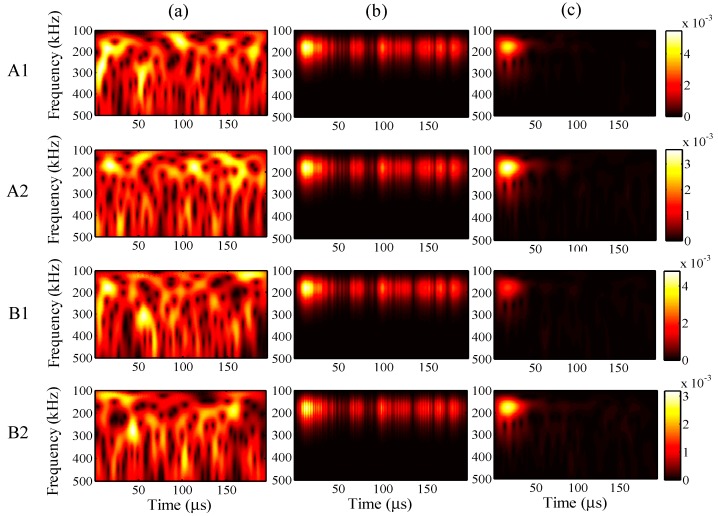
Wavelet transforms for the analysis period of 195 µs at AWG ports A1, A2, B1, and B2: (**a**) one-time measured signal (left); (**b**) one-time restored signal (middle); (**c**) 1024-time averaged signal (right).

For the different analysis periods, the results of the relative measuring error *Err*^m^ and relative analysis error *Err*^p^ are presented in Figure 17, when the maximum input amplitude is 58 µε and the input frequency is 200 kHz. Furthermore, for an analysis period of 75 µs, the frequency distribution of the wavelet transforms at AWG ports A1, A2, B1, and B2 are presented in Figure 18, for the one-time measured signal (left), one-time restored signal (middle), and 1024-time averaged signal (right). Likewise, for the analysis periods of 105, 135, and 165 µs, the results of the wavelet transforms are presented in Figure 19, Figure 20 and Figure 21, respectively.

In Figure 17, the relative errors are presented in 30-μs intervals from 45 μs. For the analysis period of 165 μs, the relative analysis error could not be assessed. In comparison with the relative measuring errors, the relative analysis errors were remarkably reduced using the proposed method. For example, the relative measuring error is 205% but the relative analysis error is only 73% in the case of 45 μs. This indicates that the noisy elements of the one-time measured signal were substantially filtered by the PARAFAC-based processing. Meanwhile, the relative measuring errors increased with an increase in the analysis period. It is thus clear that noise existed throughout the time period, and the measurement of the FBG sensors was strongly disturbed by such noise.

The relative analysis errors also increased because of the effect of such noise; however, the increasing tendency is apparently gradual, which indicates that the PARAFAC method is robust in dealing with such noise. On the other hand, the risk of missing important atoms becomes high, while the relative measuring error becomes high enough for the one-time measured signal; e.g., for the analysis period of 165 μs, paired antiphase atoms could not been found for feature selection to restore the one-time measured signal, which may result from the high relative measuring error around 400%.

In Figure 18, Figure 19, Figure 20 and Figure 21, it is clear that significant signals were detected for the one-time restored and 1024-time averaged signals, and their wavelet transforms are basically distributed around a frequency of 200 kHz. However, for the 1024-time averaged signals, the frequency distributions were not revealed after about 40 µs, which means that the conventional averaging method failed to detect weakly significant signals with longer analysis periods. Figure 20c clearly shows that, for port B2 and an analysis period of 135 µs, no significant signal can be distinguished from the 1024-time averaged signal. Conversely, for the one-time restored signals, significant signals are clearly observed even though the frequency distributions disappeared in many places. The PARAFAC-based method, compared with the conventional averaging method, is thus considered to be strongly robust against variations in the analysis period.

#### 3.3.3. Input Signal Frequency

In the former sections, the effectiveness of the proposed method was discussed in the case of an input signal having a frequency of 200 kHz. To evaluate the effectiveness for different input frequencies, the one-time measured signal was processed at input frequencies of 60, 90 and 300 kHz. Here, the maximum input amplitude is 48 με and the analysis range is 10 to 500 kHz. The analysis periods are 150, 100, and 30 μs for input frequencies of 60, 90, and 300 kHz, respectively. The analysis periods were decided as three times the input times of the signals from the MFC actuator.

**Figure 22:**
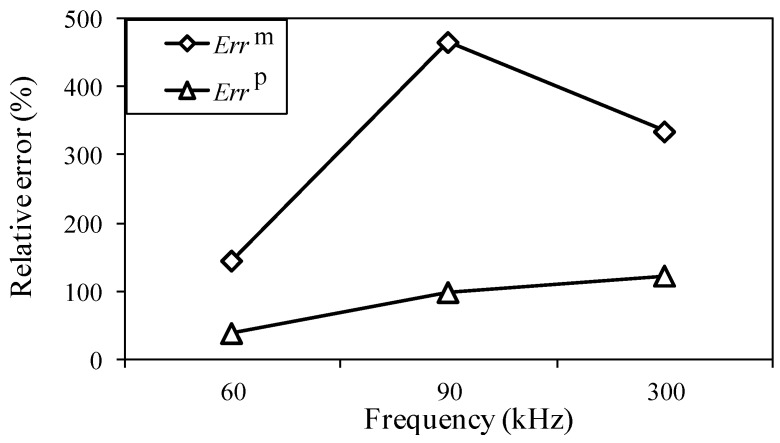
Relative measuring error *Err*^m^ and relative analysis error *Err*^p^ for different input frequencies.

The results of the relative measuring error *Err*^m^ and relative analysis error *Err*^p^, for the different the input frequencies are presented in Figure 22. With an increase in input frequency, the relative measuring errors fluctuate for the 90-kHz input frequency but the relative analysis errors gradually increase. The figure clearly shows that the signal-to-noise ratios improved for the one-time restored signals.

**Figure 23:**
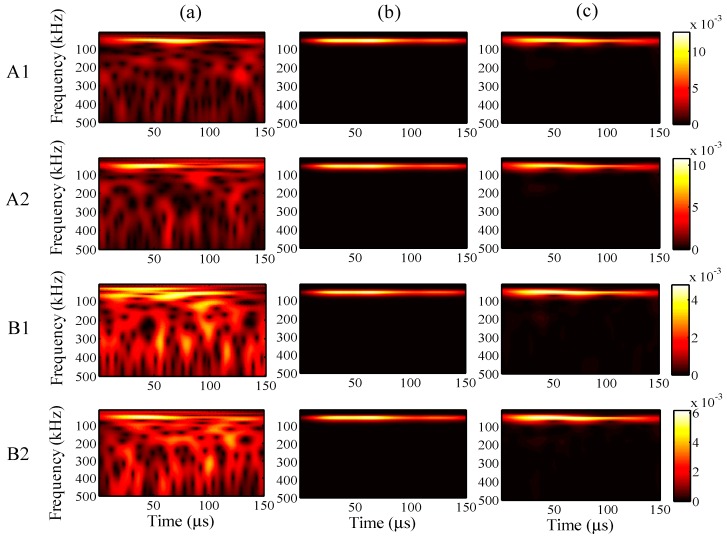
Wavelet transforms for an input frequency of 60 kHz at AWG ports A1, A2, B1, and B2: (**a**) one-time measured signal (left); (**b**) one-time restored signal (middle); (**c**) 1024-time averaged signal (right).

**Figure 24:**
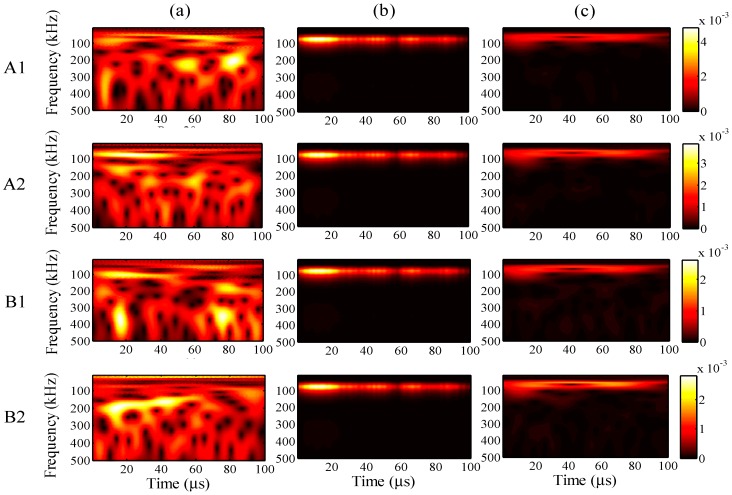
Wavelet transforms for an input frequency of 90 kHz at AWG ports A1, A2, B1, and B2: (**a**) one-time measured signal (left); (**b**) one-time restored signal (middle); (**c**) 1024-time averaged signal (right).

**Figure 25:**
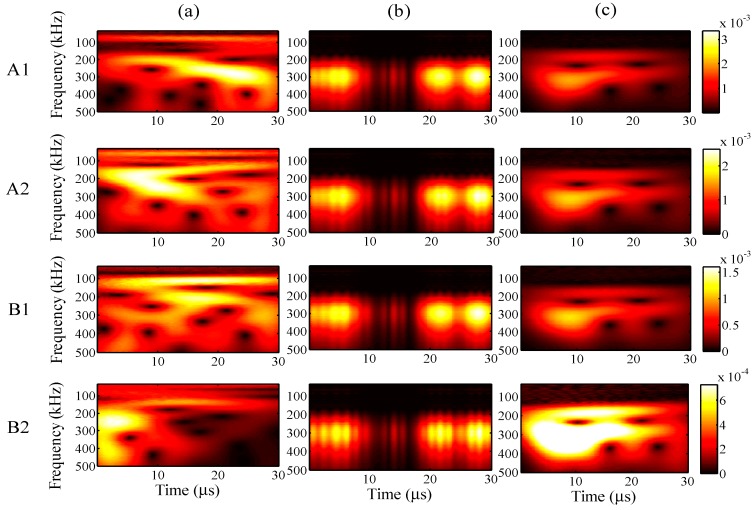
Wavelet transforms for an input frequency of 300 kHz at AWG ports A1, A2, B1, and B2: (**a**) one-time measured signal (left); (**b**) one-time restored signal (middle); (**c**) 1024-time averaged signal (right).

In the case of the input frequency of 60 kHz, the frequency distribution of the wavelet transforms at AWG ports A1, A2, B1, and B2 are presented in Figure 23a–c, for the one-time measured signal (left), one-time restored signal (middle), and 1024-time averaged signal (right). Likewise, for input frequencies of 90 and 300 kHz, the results of the wavelet transforms are presented in Figure 24 and Figure 25.

In Figure 23, strong consistency is observed between the wavelet transforms of the one-time restored and 1024-time averaged signals, regardless of the AWG port. Moreover, this consistency is also found in Figure 24, between the wavelet transforms of the one-time restored and 1024-time averaged signals. However, there is a small break close to 60 μs in Figure 24b, for the wavelet transforms of the one-time measured signals.

In Figure 25b for the one-time restored signals, the frequency distributions of the wavelet transforms were separated into two parts of 0–10 and 20–30 μs. However, in Figure 25c for the 1024-time averaged signals, the frequency distributions of the wavelet transforms were separated into two parts around 200 and 300 kHz in the vertical direction. In both of the situations, the main frequency distribution is concentrated around 300 kHz.

## 4. Conclusions

The present study investigated the signal recognition of ultrasonic waveforms by application of FBG sensors and AWG filters, for which a novel method was proposed to distinguish a significant signal from one-time measured ultrasonic waves. The method based on PARAFAC decomposition is effective in dealing with noise. Relative to the traditional averaging method, which requires thousands of repeated measurement for noise reduction, the one-time measurement method is simple and cost effective. Thereby, the proposed method employing PARAFAC decomposition for the one-time measured signal is a promising and meaningful tool for practical application.

Researchers are always seeking new hardware and design to achieve real-time detection of ultrasonic waves, normally expensive and complex. Recently, researchers did propose several sensing systems with high sensitivity that can real-time detect ultrasonic signals but sacrifice the multiplexing ability in FBG-AWG system [9,10]. However, the proposed method in this manuscript not only has real-time detection ability, but also maintains the possibility of multiplexing. This was considered one of the biggest advantages compared to the other techniques mentioned above. Based on PARAFAC decomposition, the proposed signal processing method even has the possibility of being used in other optical fiber sensing systems, when two or more correlation signals could be obtained. This method is especially suitable to the FBG-AWG system, regardless of the amount of FBG sensors. For example, it is also available for one FBG sensor with the AWG system.

The paper started with a description of an experimental system of an ultrasonic wave sensing system that mainly comprises an MFC actuator, two FBG sensors, and an AWG filter. A novel signal processing procedure was then detailed. In a series of measurement experiments conducted under different conditions, a comprehensive analysis was performed to validate the proposed signal processing strategy. Several conclusions are drawn from the results of the study:

(1) The study established a signal processing strategy that improves the signal-to-noise ratio of the one-time measured ultrasonic signal; meanwhile, a sound mathematical model was given to describe the signal processing procedure, which mainly includes complex wavelet transformation, PARAFAC decomposition, and relative error evaluation.
(2) The experimental investigation validated that the signal-to-noise ratio for a one-time measured signal can be improved through a comparison of relative measurement and relative analysis errors for different input amplitudes, analysis periods, and input frequencies of the ultrasonic wave signals. The relative measuring errors increased greatly, whereas the relative analysis errors increased gradually following increases in the analysis period and input frequency and decreases in the input amplitude.
(3) All frequency distributions of wavelet transforms were demonstrated for the one-time measured signals, one-time restored signals, and 1024-time averaged signals. It was validated that the proposed method is applicable and reliable for most experimental conditions.
